# Iron overload regulates cognitive function in rats with minimal hepatic encephalopathy by inducing an increase in frontal butyrylcholinesterase activity

**DOI:** 10.3389/fnagi.2024.1447965

**Published:** 2024-09-27

**Authors:** Hua Lan, Xuhong Yang, Minxing Wang, Minglei Wang, Xueying Huang, Xiaodong Wang

**Affiliations:** ^1^General Hospital of Ningxia Medical University, Yinchuan, China; ^2^Nurturing Center of Jiangsu Province for State Laboratory of AI Imaging and Interventional Radiology, Department of Radiology, Zhongda Hospital, Medical School, Southeast University, Nanjing, China; ^3^School of Clinical Medicine, Ningxia Medical University, Yinchuan, China

**Keywords:** minimal hepatic encephalopathy, butyrylcholinesterase, quantitative susceptibility mapping, iron deposition, cognitive function

## Abstract

**Background and aims:**

This study aimed to investigate the effect of iron overload on acetylcholinesterase activity in the frontal lobe tissue of rats with minimal hepatic encephalopathy (MHE) and its relation to cognitive ability. By elucidating the potential mechanisms of cognitive impairment, this study may offer insights into novel therapeutic targets for MHE.

**Materials and methods:**

Twelve Sprague-Dawley rats were purchased and randomly assigned to either the experimental or control group with six rats in each group. Following the induction of MHE, the Morris Water Maze (MWM) was utilized to assess spatial orientation and memory capacity. Subsequently, Magnetic Resonance Imaging (MRI) scans were performed to capture Quantitative Susceptibility Mapping (QSM) images of all rats' heads.

**Results:**

Compared to the control group rats, the MHE model rats showed significantly reduced learning and memory capabilities as well as spatial orientation abilities (*P* < 0.05). Furthermore, the susceptibility values in the frontal lobe tissue of MHE model rats was significantly higher than that of the control group rats (*P* < 0.05), and the corresponding BuChE activity in the frontal lobe extract of model rats was significantly increased while BuChE activity in the peripheral blood serum was significantly decreased compared to the control group rats (*P* < 0.05). Meanwhile, our findings indicate a significant positive correlation between latency period and BuChE activity with susceptibility values in the MHE group.

**Conclusion:**

The changes in BuChE activity in frontal lobe extract may be related to changes in spatial orientation and behavioral changes in MHE, and iron overload in the frontal lobe tissue may regulate changes in BuChE activity, BuChE levels appear to be iron-dependent.

## 1 Introduction

Hepatic encephalopathy (HE) is a frequent complication characterized by cognitive decline and abnormal neurologic function, with high prevalence worldwide (Bernal et al., 2010). HE results from liver disease-induced neurological and psychiatric disorders, leading to minimal hepatic encephalopathy (MHE), a mild form of HE. As MHE progresses, patients display defects in attention, motor function, and arousal (Hadjihambi et al., 2018; Khungar and Poordad, 2012; Gairing et al., 2023). Up to 56% of MHE patients will develop severe neurological and psychiatric syndromes within 3 years, resulting in irreversible cognitive impairment (Weissenborn et al., 2004). Hence, early diagnosis and treatment of MHE are critical for improving patient outcomes. Nevertheless, the pathophysiological mechanisms underlying MHE remain unclear.

Recent studies have linked defects in the brain neurotransmitter system, particularly glutamate and monoaminergic mechanisms, gamma-aminobutyric acid (GABA), and serotonin systems, to the occurrence of MHE in animal models and liver disease patients (Hamdani et al., 2021; Dasarathy and Mullen, 1998; Yurdaydin et al., 1996). However, there have been relatively few reports on changes in the cholinergic system in MHE. One study confirmed direct evidence that liver failure leads to an imbalance in the brain cholinergic system by detecting changes in acetylcholinesterase (AChE) activity in brain tissue in an animal model (Garcia-Ayllon et al., 2008), but no meaningful conclusion has been reached regarding whether butyrylcholinesterase (BuChE) participates in the development of MHE-related liver damage and cognitive impairment.

The cholinergic system plays a crucial role in specific behavioral responses and cognitive processes (Berlot et al., 2021). AChE in cerebrospinal fluid is the principal enzyme regulating acetylcholine (ACh) levels, promptly degrading the neurotransmitter of cholinergic synapses. In contrast, BuChE in human plasma exhibits low specificity and insignificant physiological effects in regulating the cholinergic system (Hall, 1973). Nonetheless, some studies have demonstrated that BuChE's role in regulating the cholinergic system is critical (Duysen et al., 2007; Mesulam et al., 2002). It collaborates with AChE to jointly degrade neurotransmitter ACh and participate in cholinergic regulation. One study detected the activity of peripheral BuChE and red blood cell (RBC) count in 1,200 people and found a robust correlation between BuChE levels and RBC count (Jasiecki et al., 2016). They hypothesized that BuChE levels might be iron-dependent because RBC binds 65% of the body's iron. Furthermore, researchers discovered an iron response element in the mRNA sequence of glial cell BuChE's 30-UTR, indicating that BuChE may be regulated by iron homeostasis (Campillos et al., 2010), and previous studies have confirmed changes in brain tissue iron content in MHE (Liu et al., 2013; Yang et al., 2022).

Therefore, this study aims to explore potential changes in blood serum and brain tissue BuChE activity and its relationship with cognitive function under the background of MHE in model rats and determine whether BuChE activity changes are related to iron overload. We hypothesize that abnormal iron accumulation in the MHE brain region induces increased BuChE expression, which consumes the neurotransmitter ACh through a bypass pathway, thereby accelerating cognitive impairment in the MHE brain cholinergic system.

## 2 Materials and methods

### 2.1 Subjects

**Scheme 1:**
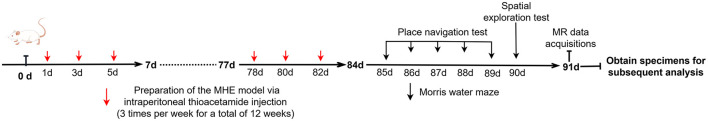
The timeline for the entire experimental process.

Twelve Sprague-Dawley rats (6–8 weeks, 200–260 g) were purchased from the Experimental Animal Center of Ningxia Medical University. After 1 week of acclimation, the animals were randomly assigned to experimental (*n* = 6; MHE group) and control (*n* = 6; HC group) groups, and housed in standard laboratory conditions (20–22°C, 65%−70% relative humidity, 12-hour light/dark cycle). All procedures in this study were approved by the Animal Research Committee of Ningxia Medical University (NO: IACUC- NYLAC-2021-114). Based on the recommendations of the International Society for Hepatic Encephalopathy and Nitrogen Metabolism (ISHEN), thioacetamide (TAA) was used to induce MHE (Farjam et al., 2012). TAA can produce a predictable and reliable dose- and time-dependent liver injury model similar to human fibrosis and cirrhosis (Al-Bader et al., 2000). In this study, the experimental group received TAA at a starting concentration of 150 mg/kg (three times per week for a total of 12 weeks) to induce MHE, while the control group received an equivalent dose of physiological saline via intraperitoneal injection. The timeline for the entire experimental process is as shown in Scheme 1.

### 2.2 Morris water maze test all rats

The Morris water maze (MWM) consists of a circular pool measuring 200.0 cm × 200.0 cm × 55.0 cm, filled with water to a depth of 37.0 cm with an escape platform located 2.0 cm below the water surface. The water temperature was maintained at 22 ± 2°C. To minimize external factors' potential confounding effects on the experimental animals, the pool was surrounded by canvas, whilst plastic boards with varying shapes were affixed to the walls in the east, south, west, and north directions to assist the rats in orientating themselves and determining their location.

Place navigation test: The experiment consisted of 5 days, with four training sessions per day. At the start of each session, rats were introduced into the water from any point on the wall facing the pool. Their swimming trajectories and times were recorded using a SMART 3.0 computer video tracking system (Panlab, Spain). The average score of the four daily training sessions was used to evaluate the learning performance of the rats. Throughout the experiment, all rats had to locate and remain on the escape platform for 10 s within a 60-s timeframe. If the rat failed to reach the platform within 60 s, it was placed on the platform for 10 s before the next trial.

Spatial exploration test: On the sixth day, the escape platform was removed, and all rats were allowed to swim freely in the MWM for 60 s. The SMART 3.0 computer video tracking system automatically recorded the behavioral indices of the rats in the maze (time spent in the target quadrant and number of times crossing the target quadrant), and the behavioral performance of all rats was observed.

### 2.3 MR data acquisitions

On the day of MRI data acquisition, isoflurane (induction 5%, maintenance 2%, oxygen flow rate 1.5 L/min) was utilized to anesthetize the rats. Subsequently, all rats underwent imaging using a GE Architect 3.0T MR scanner equipped with a dedicated rat coil. To prevent image quality degradation and motion artifacts caused by head movement during scanning, the tails and heads of the rats were secured using medical tape and sponge pads before the experiment. The rats' condition was continuously monitored throughout the scan. Before the QSM scans, routine sequence scans were performed to exclude rats with brain lesions. QSM data acquisition was performed using a 3D gradient-echo (GRE) sequence with the following parameters: TR/TE = 100.4 ms/8 ms, flip angle = 12°, slice thickness = 0.7 mm, slice spacing = 1.0 mm, FOV = 80 × 80 mm.

Data post-processing: The raw images were transferred to a personal computer in DICOM format using RadiAnt DICOM Viewer software (https://www.radiantviewer.com/). The STI_Suite_V3.0 software was utilized to obtain amplitude, phase, and QSM images, following which the processed QSM images were saved in “NII” format for further analysis. The “NII” files were imported into ITK-SNAP_3.8 software (http://www.itksnap.org/) to draw regions of interest (ROIs) and measure the susceptibility values (Li et al., 2020). To reduce errors, the maximum level of brain tissue in the axial plane and the two adjacent planes above and below it were selected for ROI drawing. Three ROIs were drawn on each plane, the average value of which from the three planes was used as the final value.

### 2.4 Enzyme-linked immune-sorbent assay

The day after the QSM data acquisition, blood was drawn from the tail vein to assess the RBC count (BC-2800vet, Mindray, China). Rats were euthanized after blood collection, and frontal cortex and liver tissues were rapidly harvested on ice. Fix the frontal cortex and liver tissue with 4% paraformaldehyde, stain with iron, and quantify iron deposits using Eclipse Ci-L microscopy and Image Pro Plus 6.0 (Media Cybernetics, Silver Spring, MD, USA). Calculate iron deposition as the pixel area of iron ion precipitation/field of view pixel area ^*^ 100. For the quantification of BuChE activity in rat serum and brain tissue, we utilized a commercially available ELISA kit (A025-1, Nanjing Jiancheng, China).

Subsequently, the tissue sample was subjected to a wash step with pre-chilled phosphate-buffered saline (PBS; 0.01 M, pH = 7.4) to eliminate residual blood content before being weighed. Next, physiological saline solution (0.9%) was added to the tissue sample at a predetermined weight-to-volume ratio of 1:9. Afterward, the sample underwent mechanical homogenization in an ice-cold water bath at 2,500 rpm for 10 min. The supernatant containing the soluble components was collected for further experimentation as per the instructions stipulated by the manufacturer.

### 2.5 Statistical analysis

Statistical analyses were performed using SPSS-26.0 and GraphPad Prism 9.5 software (GraphPad Software, USA). Prior to statistical testing, we assessed the normality of all data using the Kolmogorov–Smirnov test. For normally distributed continuous variables, we employed the *Student's t-test* to determine differences between groups and reported our findings as means ± standard deviation (*x* ± s). Conversely, for non-normally distributed continuous variables, we conducted the *Mann–Whitney U*-test and presented the results as median ± interquartile range [*M* (Q1, Q3)]. We utilized either Pearson or Spearman correlation analysis to investigate relationships between individual parameters. Significance was established at *P* < 0.05.

## 3 Result

### 3.1 Latency period in the MWM experiment in rats

We evaluated the temporal changes in spatial learning ability in all rats using the place navigation test. A statistically significant reduction in latency period (time to reach the original platform) was observed in the HC group as the number of training sessions increased, with a significant difference noted between the fourth and fifth days (*P* = 0.005; Table 1). In contrast, the MHE group exhibited time-dependent spatial learning impairments compared to MHE group. These findings suggest that compared to the HC group, the MHE group had significantly reduced spatial learning ability.

**Table 1:** MWM latency time of rats in two groups.

**Groups**	**HC group (*n* = 6)**	**MHE group (*n* = 6)**	***t-*value**	***P*-value**
Day 1 (s)	56.40 ± 5.29	57.96 ± 5.22	−0.516	0.617
Day 2 (s)	35.27 ± 7.32	45.13 ± 12.57	−1.660	0.128
Day 3 (s)	26.37 ± 3.90	37.19 ± 12.05	−2.090	0.063
Day 4 (s)	19.94 ± 6.92	34.78 ± 7.36	−3.596	0.005
Day 5 (s)	13.30 ± 5.21	30.27 ± 10.42	−3.565	0.005

**Figure 1:**
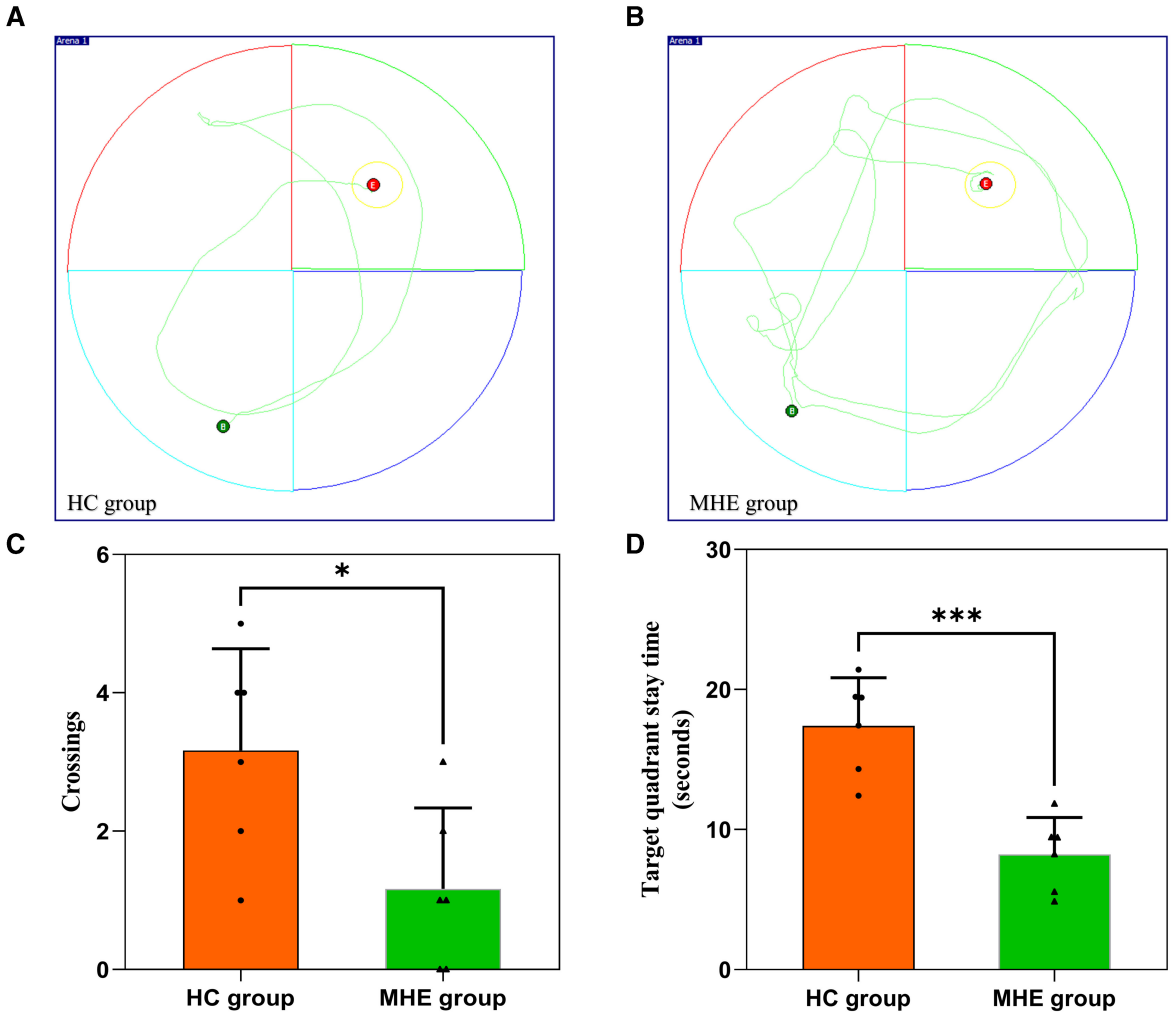
Spatial memory performance of HC and MHE groups. **(A, B)** The representative trace of rats in the MWM; **(C)** The number of times that the rat crosses the platform in the space exploration experiment; **(D)** Target quadrant stay time. *p < 0.05 and ***p < 0.001.

On the sixth day of the experiment, we conducted a spatial probe test to assess the rats' spatial orientation and memory abilities. We found that the MHE group had significantly fewer crossings than the HC group (Figure 1C; *P* = 0.026). Furthermore, the MHE group spent significantly less time in the target quadrant than the HC group (Figure 1D; *P* < 0.001). Collectively, these results suggest that compared to the HC group, the MHE group had significantly reduced spatial orientation and memory abilities, indicative of cognitive deficits.

### 3.2 BuChE activity in rat serum and frontal lobe tissue

**Figure 2:**
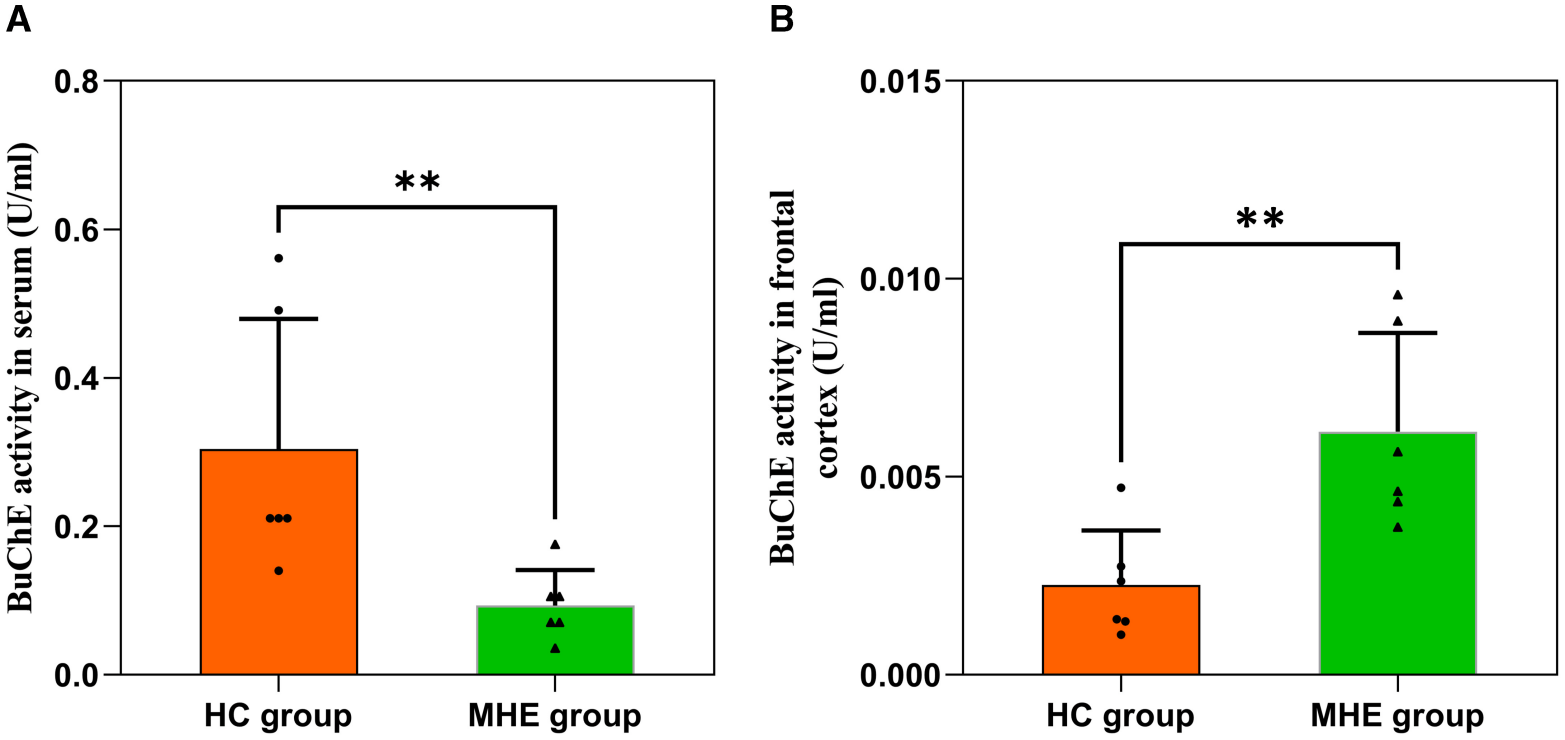
BuChE activity in the peripheral and central of HC and MHE groups. **(A)** BuChE activity in serum; **(B)** BuChE activity in frontal lobe tissue. **p < 0.01.

We assessed BuChE activity in the serum and frontal lobe tissue of HC and MHE groups. Our results show that BuChE activity in the serum of MHE group was significantly lower than that of the HC group (Figure 2A; *P* = 0.004), while BuChE activity in the frontal cortex of MHE group was significantly higher than the HC group (Figure 2B; *P* = 0.009).

### 3.3 Susceptibility values in the frontal lobe tissue of rats

**Figure 3:**
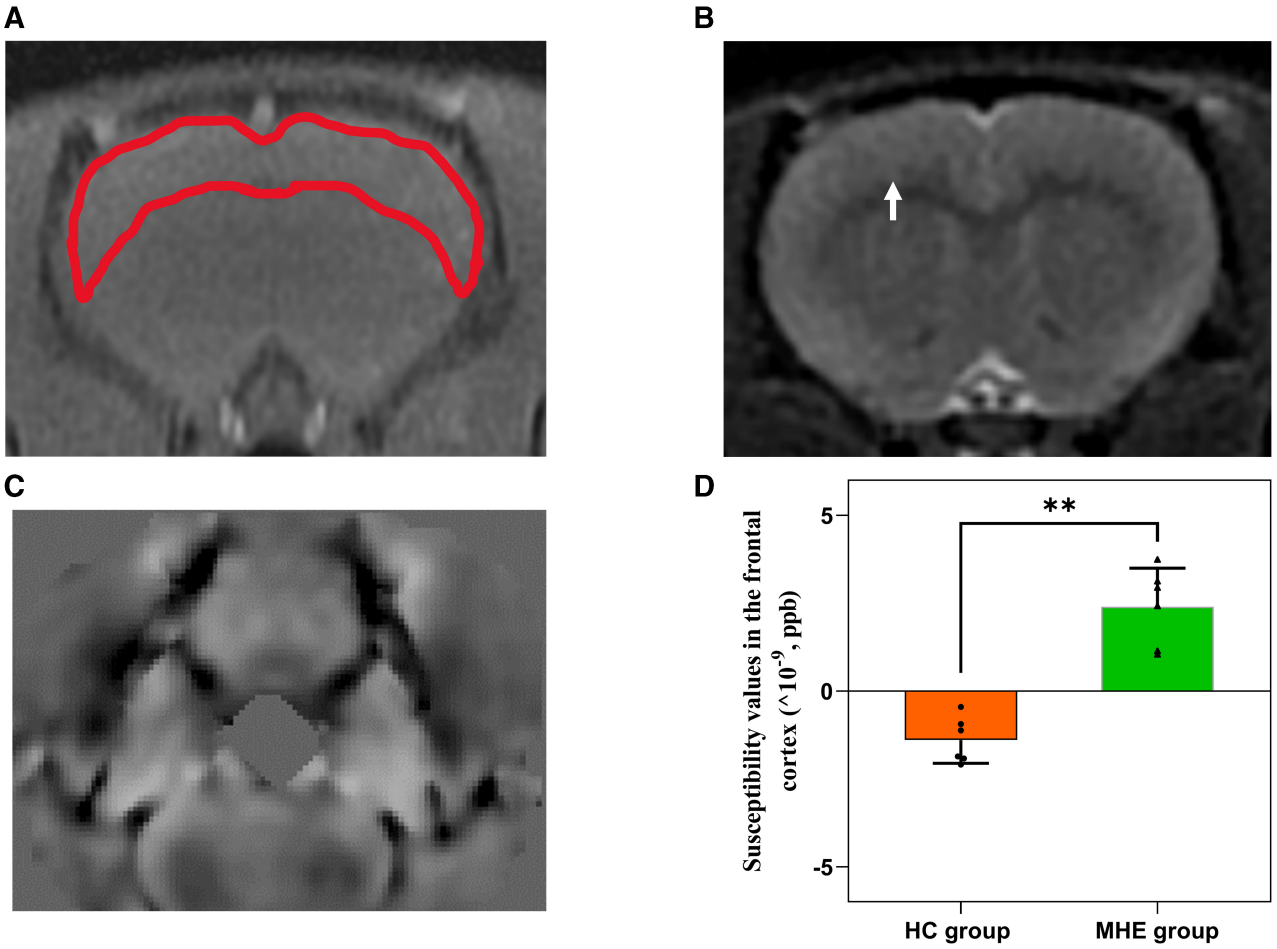
The frontal lobe susceptibility values of HC and MHE groups. **(A)** T1WI; **(B)** T2WI (arrows indicate rat frontal lobe); **(C)** QSM; **(D)** The difference in susceptibility values of frontal lobe between MHE and HC groups. **p < 0.01.

Figure 3 summarizes the susceptibility values in the frontal cortex of both groups of rats. We found that the susceptibility values in the frontal cortex of MHE group was significantly higher than that of the HC group (*P* = 0.002). These observations suggest that iron content is increased in the frontal cortex of MHE group, indicating abnormal iron deposition associated with MHE.

### 3.4 Assessment of iron deposition

**Figure 4:**
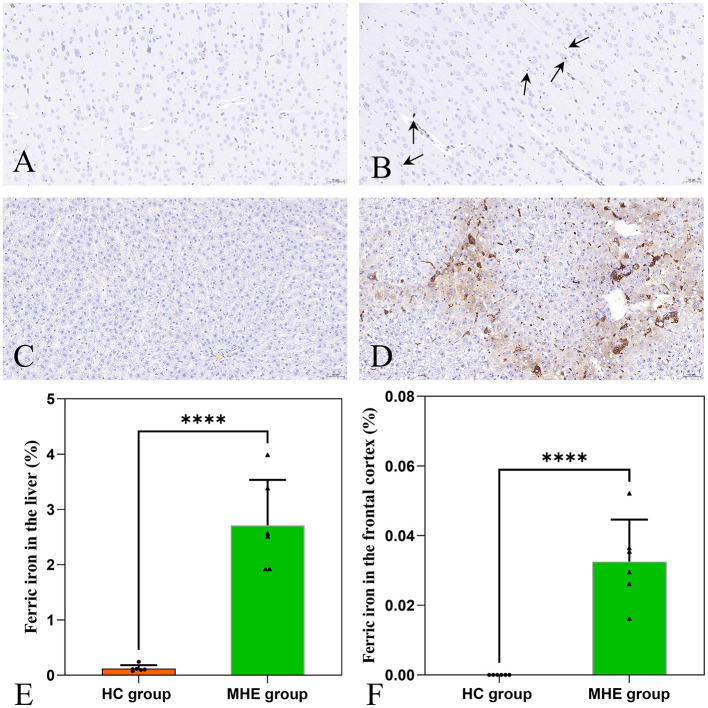
DAB-enhanced Prussian blue staining of HC and MHE groups. **(A)** Prussian blue staining of the frontal cortex of HC group; **(B)** Prussian blue staining of the frontal cortex of MHE group (the arrow indicates iron ion); **(C)** Prussian blue staining of liver tissue in HC group; **(D)** Prussian blue staining of liver tissue in MHE group; **(E)** The proportion of iron ion deposition area in the liver, **(F)** The proportion of iron ion deposition area in frontal cortex. ****p < 0.0001.

Brown granules indicative of iron was visible in the MHE frontal cortex stained with DAB-enhanced Prussian blue (Figure 4B) while no such granules were seen in HC group (Figure 4A). This indicates the presence of abnormal iron deposition in the MHE group. Furthermore, large numbers of iron-associated brown granules were also observed in MHE group liver (Figure 4D), with only small amounts of iron seen in HC group (Figure 4C). These observations suggest disordered peripheral iron metabolism associated with MHE group. Analysis of the areas of iron deposition using Image-Pro Plus showed significantly higher iron deposition in both the frontal cortex and livers of the MHE group compared with the HC group (Figures 4E, F; *P* < 0.0001).

### 3.5 RBC count and BuChE levels in serum

**Figure 5:**
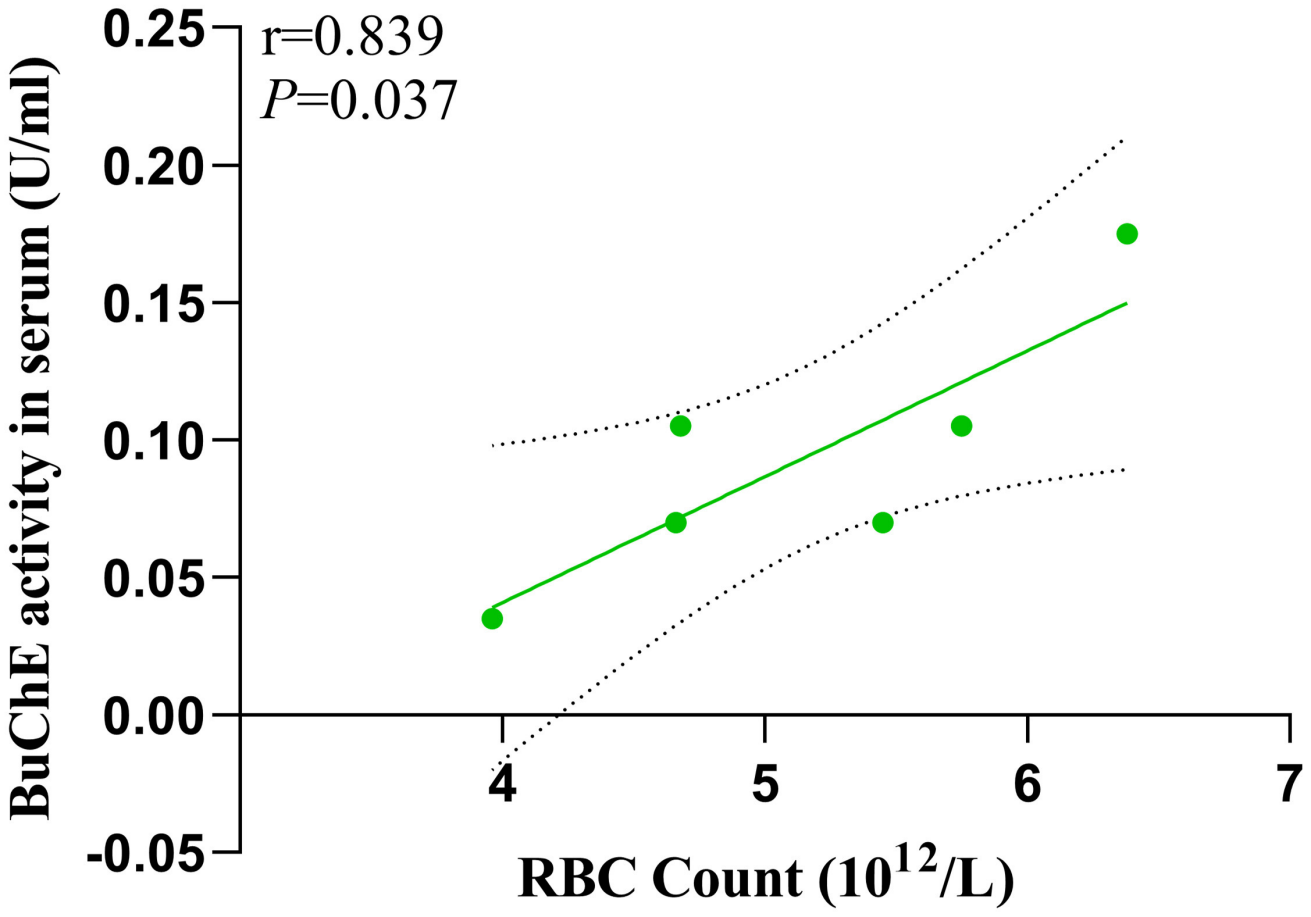
Interrelationship of BuChE activity in serum and RBC count.

In the MHE group, we found a significant positive correlation (*P* = 0.037) between serum BuChE activity and peripheral RBC count (Figure 5).

### 3.6 Correlation between susceptibility values and target quadrant stay time

**Figure 6:**
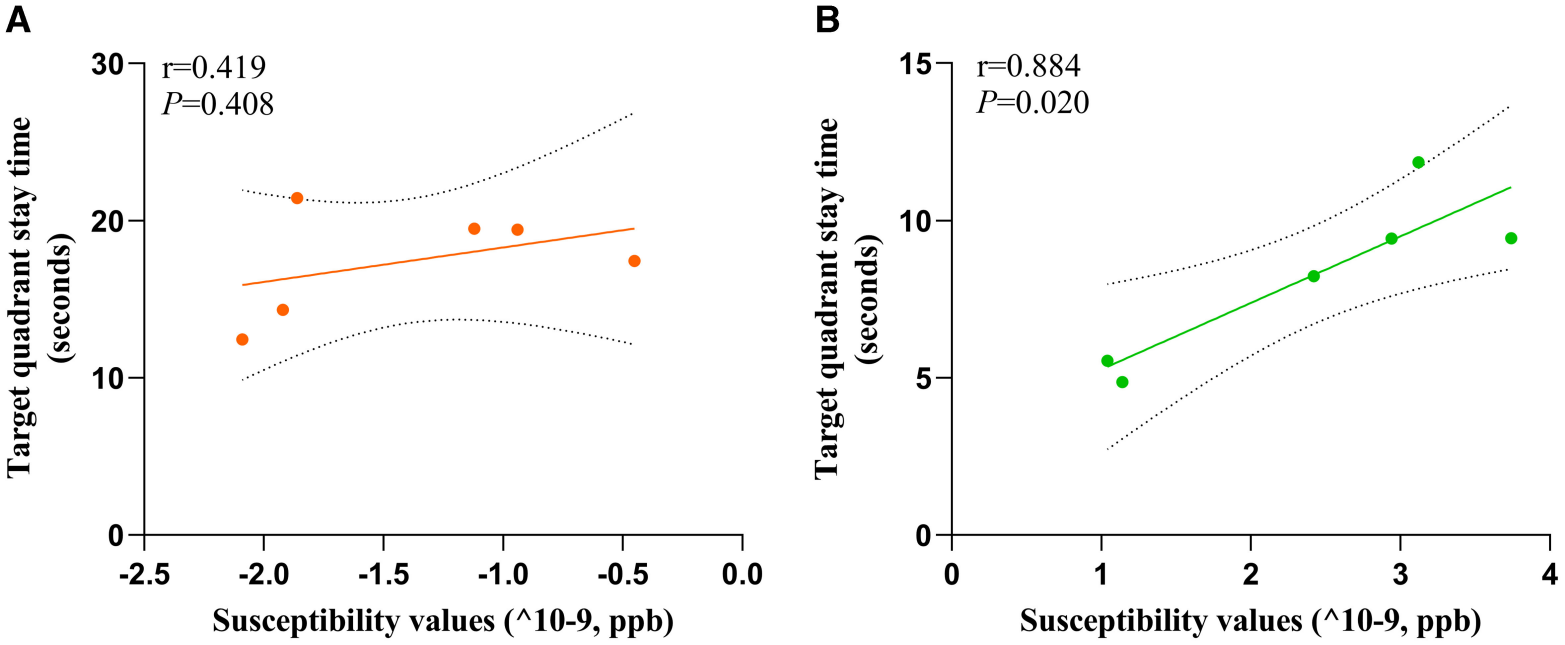
Correlation analysis was used to predict the relationship between spatial memory and susceptibility values of frontal cortex in all rats. Correlation analysis of **(A)** and **(B)**. Target quadrant stay time and susceptibility values; **(A)** HC group; **(B)** MHE group.

To investigate the potential relationship between cognitive impairment and iron accumulation, we conducted a correlation analysis between the target quadrant stay time in spatial exploration test (on the sixth day) of HC and MHE groups with susceptibility values in the frontal cortex. Our results revealed a significant positive correlation between the susceptibility values and the latency period in the MHE group (*r* = 0.844, *P* = 0.020, Figure 6B). However, no such correlation was observed in the HC group (*r* = 0.419, *P* = 0.408, Figure 6A). These findings suggest that there may be a direct relationship between susceptibility values and cognitive impairment associated with iron accumulation in the brain.

### 3.7 Relationship between susceptibility values and BuChE activity of rats

**Figure 7:**
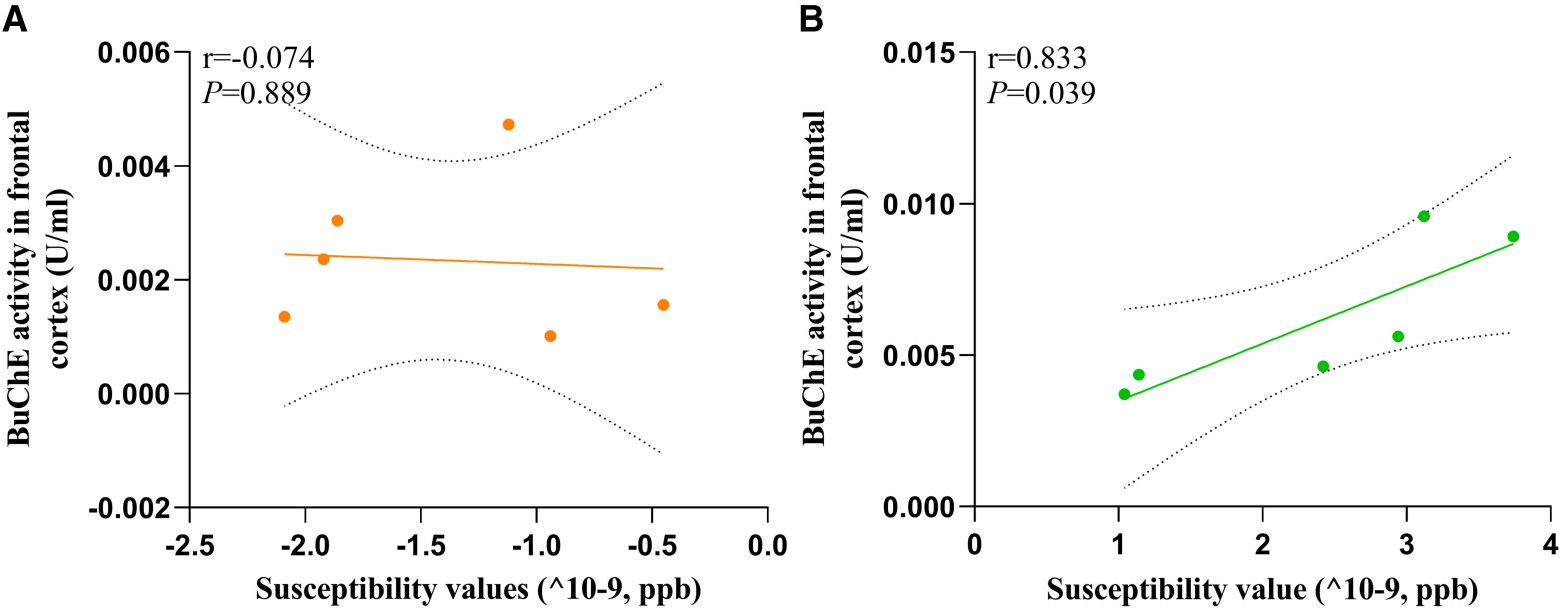
The correlation between the BuChE activity and susceptibility values of frontal cortex in all rats; **(A)** HC group; **(B)** MHE group.

To evaluate the relationship between susceptibility and BuChE activity, we conducted a correlation analysis between these variables in the frontal cortex of HC and MHE groups. Our analysis revealed a significant positive correlation between susceptibility values and BuChE activity (*r* = 0.833, *P* = 0.039, Figure 7B) in MHE group, whereas no such correlation was observed in HC group (*r* = −0.074, *P* = 0.889, Figure 7A).

## 4 Discussion

This study utilized QSM technology to evaluate alterations in iron content within the frontal lobe tissue of rats with MHE. Our findings revealed a significant increase in susceptibility values within the bilateral frontal lobe tissue of MHE group. Additionally, we observed a noteworthy enhancement in BuChE activity levels in the frontal cortex of MHE group, while BuChE activity within their serum significantly decreased. Further analysis demonstrated a significant correlation between susceptibility values and BuChE activity in the frontal cortex of MHE group. Furthermore, our research determined a positive correlation between BuChE activity in the peripheral blood of MHE rats and their RBC count. These results may provide insight into the underlying biological mechanisms associated with cognitive impairment in MHE and emphasize the potential role of iron overload in this disease.

In this study, a murine model of MHE was established by utilizing TAA. Cognitive impairments such as deficits in memory, learning, and spatial orientation were evaluated in the MHE group through MWM testing. Due to its demonstrated accuracy in measuring memory and attention deficits (Singh and Trigun, 2014), MWM testing allowed for the prediction of mild levels of attention and memory deficits in the model rats. The research findings disclosed that MHE group exhibited memory impairment after the fourth day of the training period (Table 1; Figure 1), specifically encompassing (1) an extended latency to reach the platform, (2) fewer crossings of the target quadrant, and (3) reduced time spent in the target quadrant. These outcomes indicate that when compared with HC group, the MHE model rats displayed significantly decreased abilities in learning, memory, and spatial orientation.

Iron is the most abundant metal in the human body and plays a critical role in numerous biological processes (Yang et al., 2018). However, an excess of reactive iron within cells can result in cytotoxicity through multiple mechanisms, such as oxidative stress and the promotion of lipid peroxidation (Stockwell et al., 2017; Heneka et al., 2015). Recently, various studies have demonstrated that excessive iron levels in the brain play a pivotal role in cognitive impairment (Yang et al., 2022; Sun et al., 2017). In this study, we utilized QSM to assess the brain iron levels of all subjects. The findings indicated a significant increase in susceptibility values within the frontal lobe tissue of MHE model rats, suggesting abnormal iron deposition compared to the HC group. Brown granules indicative of iron was also visible in the MHE frontal cortex stained with DAB-enhanced Prussian blue, simultaneously, a significant accumulation of iron ions was noted in the liver of the MHE group. The central nervous system's uptake of iron is a complex and highly regulated process (Ward et al., 2014). In this study, we speculate that in the onset and development of MHE, liver pathology leads to changes in hepcidin levels, causing an imbalance in peripheral iron homeostasis, ultimately resulting in abnormally elevated peripheral iron levels. The increase in peripheral iron levels facilitates the passage of iron through the endothelial cells lining the blood-brain barrier, leading to an increase in brain iron content. To establish a potential correlation between cognitive impairment in the MHE group and brain iron deposition, we conducted a correlation analysis of the latency period (on the sixth day) during the location navigation experiment with the susceptibility values observed in the frontal lobe tissue of the MHE group. Our results demonstrated a significant positive correlation between the latency period and susceptibility values in the MHE group (Figure 6). The latency period reflects the cognitive level of the MHE group, while the susceptibility values indirectly reflect the iron level in the frontal lobe tissue of the group. Consequently, based on the above data and results, we suggest that brain iron overload may represent one of the initial causes of cognitive impairment in the MHE group, while peripheral iron disorder may potentially lead to central iron deposition.

Cholinergic neurons are primarily located in areas of the brain that are associated with cognitive function (Méndez et al., 2011). The normal cholinergic signaling pathway that is linked to cognitive function relies on the neurotransmitter ACh. ACh serves as the primary neurotransmitter synthesized by cholinergic neurons, and AChE plays a crucial role in depleting cholinergic neurotransmitters. Interestingly, studies have reported the expression of BuChE in various regions of the human brain, including the amygdala, hippocampus, frontal lobe, and thalamus (Darvesh et al., 1998), suggesting its potential role in cognitive function due to its unique distribution within the central nervous system and expression in structures involved in cognition. Previous research has demonstrated that mice can survive without AChE due to the presence of BuChE, which hydrolyzes ACh when AChE is absent (McArdle et al., 2005). In this study, we found significant increases in BuChE activity within the frontal lobe tissue of MHE model rats, while also observing decreased BuChE activity within their peripheral blood serum (Figure 2), which is consistent with previous reports in MHE patients. Although we acknowledge that the MHE disease model utilized in our study may not entirely replicate the complexity and variability of human MHE disease, we attempted to use a model that approximates liver injury and fibrosis processes in humans, allowing for enhanced validity of our findings. However, some reports have not observed changes in BuChE activity in brain tissue during related studies (Rao et al., 1994). We discovered that the differences in buffer solutions used in previous research, lacking detergents, may account for the discrepancies between our results and theirs. Additionally, we investigated the relationship between frontal lobe iron overload and BuChE in the MHE model rats. Our findings showed a positive correlation between susceptibility values and BuChE activity within the frontal lobe tissue of MHE model rats, while no significant differences were observed in the control group rats (Figure 7), suggesting that BuChE levels may be iron-dependent, consistent with our hypothesis.

Our study results suggest that an imbalance in the cholinergic system related to cognitive function may represent a neuropathological characteristic of MHE, with liver failure-induced iron dyshomeostasis potentially playing a predominant role in this process. Elevated iron-dependent BuChE levels can accelerate the decay of ACh in the brain, leading to a deterioration of cognitive status in MHE patients. Previous research has demonstrated significant reductions in ACh content in specific brain regions in HE model rats (Garcia-Ayllon et al., 2008). Therefore, based on our research and data, we hypothesize that BuChE and iron play crucial roles in regulating the cholinergic system in the brains of MHE patients and are associated with cognitive functions such as learning and memory.

In the context of liver failure, the disruption of iron homeostasis culminates in iron overload, which can trigger BuChE expression and accelerate the progression of MHE. Consequently, it is pertinent to investigate whether a combinatory therapeutic approach involving cholinesterase inhibitors and natural iron chelators like lactoferrin may represent a more efficacious treatment strategy for MHE.

## 5 Conclusion

This study aimed to investigate the relationship between iron overload and BuChE in MHE. Our findings demonstrated that peripheral blood levels of BuChE were positively correlated with RBC count in MHE model rats. Iron overload may act as a modulator of changes in BuChE activity observed within the frontal lobe tissue of MHE model rats, while the alterations in BuChE activity detected within the frontal lobe extract may be associated with spatial orientation and behavioral changes relating to MHE. These discoveries have provided potential therapeutic targets for MHE treatment, augmenting our understanding of the pathophysiological mechanisms underlying MHE and providing new avenues for its clinical management.

## Data Availability

The raw data supporting the conclusions of this article will be made available by the authors, without undue reservation.
